# Evaluation of human cartilage endplate composition using MRI: Spatial variation, association with adjacent disc degeneration, and in vivo repeatability

**DOI:** 10.1002/jor.24787

**Published:** 2020-07-07

**Authors:** Linshanshan Wang, Misung Han, Jason Wong, Patricia Zheng, Ann A. Lazar, Roland Krug, Aaron J. Fields

**Affiliations:** ^1^ Department of Orthopaedic Surgery University of California San Francisco California; ^2^ Department of Radiology & Biomedical Imaging University of California San Francisco California; ^3^ Department of Epidemiology and Biostatistics University of California San Francisco California; ^4^ Department of Preventive and Restorative Dental Sciences University of California San Francisco California

**Keywords:** cartilage endplate, intervertebral disc degeneration, low back pain, magnetic resonance imaging (MRI), T2*

## Abstract

Cartilage endplate (CEP) biochemical composition may influence disc degeneration and regeneration. However, evaluating CEP composition in patients remains a challenge. We used T2* mapping from ultrashort echo‐time (UTE) magnetic resonance imaging (MRI), which is sensitive to CEP hydration, to investigate spatial variations in CEP T2* values and to determine how CEP T2* values correlate with adjacent disc degeneration. Thirteen human cadavers (56.4 ± 12.7 years) and seven volunteers (36.9 ± 10.9 years) underwent 3T MRI, including UTE and T1ρ mapping sequences. Spatial mappings of T2* values in L4‐S1 CEPs were generated from UTE images and compared between subregions. In the abutting discs, mean T1ρ values in the nucleus pulposus were compared between CEPs with high vs low T2* values. To assess in vivo repeatability, precision errors in mean T2* values, and intraclass correlation coefficients (ICC) were measured from repeat scans. Results showed that CEP T2* values were highest centrally and lowest posteriorly. In the youngest individuals (<50 years), who had mild‐to‐moderately degenerated Pfirrmann grade II‐III discs, low CEP T2* values associated with severer disc degeneration: T1ρ values were 26.7% lower in subjects with low CEP T2* values (*P* = .025). In older individuals, CEP T2* values did not associate with disc degeneration (*P* = .39‐.62). Precision errors in T2* ranged from 1.7 to 2.6 ms, and reliability was good‐to‐excellent (ICC = 0.89‐0.94). These findings suggest that deficits in CEP composition, as indicated by low T2* values, associate with severer disc degeneration during the mild‐to‐moderate stages. Measuring CEP T2* values with UTE MRI may clarify the role of CEP composition in patients with mild‐to‐moderate disc degeneration.

## 1 INTRODUCTION

The biochemical composition of the cartilage endplate (CEP) is believed to be a key factor that influences intervertebral disc health. For example, nutrients entering the avascular disc and exiting metabolites must pass through the CEP,^1^ and deficits in CEP composition that result in lower water content or porosity, such as fibrosis or calcification, can block solute passage. This could hinder disc cell survival and function^2^ as well as limit the success of disc regenerative therapies that increase nutrient demands.^3^, ^4^ However, despite the theoretical importance of CEP biochemical composition, little is known about how CEP composition varies spatially within or between individuals. Likewise, the precise relationship between CEP biochemical composition and adjacent disc health also remains unclear. Characterizing the spatial variation in CEP composition and determining the relationship between CEP composition and adjacent disc health could provide insight into disc degeneration etiology and uncover diagnostic targets that may be useful for selecting ideal discs for treatment.

The biochemical composition of the CEP consists mainly of type II collagen, glycosaminoglycans (GAGs), and water.^5^ The relative quantities of these constituents vary spatially, the GAG and water contents being higher in the CEP tissue adjacent to the nucleus pulposus (NP) than in the CEP tissue adjacent to the annulus fibrosus.^6^, ^7^ The composition of the CEP also depends on age and the stage of disc degeneration. For example, during early stages of degeneration, the GAG and water contents in the central CEP decrease,^6^, ^8^ which coincides with reductions in CEP permeability^9^ that are presumed to imperil disc nutrition. However, detailed understanding of intra‐ and inter‐CEP variations in biochemical composition is lacking, owing in part to the difficulty of sampling the CEP tissues in a robust manner. And although the links between CEP composition and adjacent disc health are being uncovered, the clinical relevance is unclear. This is because the CEP is not visible with conventional magnetic resonance imaging (MRI) sequences currently used in the clinic, and thus, it is difficult to identify patients with disc degeneration in whom deficits in CEP composition may be a contributing factor.

Recent advances in MRI enable visualization of the CEP^10^, ^11^, ^12^ and noninvasive estimation of its biochemical composition.^11^ For example, T2* relaxation times derived from ultrashort echo‐time (UTE) MRI are positively correlated with CEP hydration and negatively correlated with the ratio of collagen‐to‐GAGs.^11^ Notably, both characteristics—the relative amount of collagen in the CEP and the degree of hydration or porosity—influence solute uptake^13^ and diffusion.^2^, ^7^ In the present study, we used T2* mapping to study the biochemical composition of CEP ex vivo and in vivo. We first quantified spatial variations in CEP T2* relaxation times within and between CEPs. Next, to determine how CEP composition impacts the extent of adjacent disc degeneration, we compared T1ρ relaxation times of the NP to the adjacent CEP T2* values. T1ρ values are positively correlated with the GAG and water contents of the NP,^14^ and thus, T1ρ assessment is sensitive to NP biochemical changes that typify early disc degeneration.^15^, ^16^ Last, we quantified the in vivo repeatability of CEP T2* measurements to gauge the clinical feasibility of using T2* values to noninvasively assess CEP composition.

## 2 METHODS

This study was approved by our Institutional Review Board. Informed consent was obtained from each subject.

### 2.1 Cadavers and subjects

Thirteen whole, fresh unfixed cadavers within 5 days postmortem (age: 56.4 ± 12.6 years, range: 25‐73 years) and seven healthy human subjects without prior history of back pain or spinal pathology (VAS ≤ 1; age: 36.9 ± 10.9 years, range: 26‐53 years) were studied. Major exclusion criteria for the subjects and cadavers included pregnancy, spondylolisthesis, scoliosis, prior lumbar surgery, disc herniation, and compression fracture. Additional exclusion criteria that were applied to the subjects included diabetes, positive smoking status, cancer, and active use of osteoporosis medication(s).

### 2.2 Image acquisition

Clinical MRI was performed on a Discovery MR 750 3T scanner using an 8‐channel Cervical Thoracic Lumbar Coil (GE Healthcare). Cadaver MRI was performed on a Discovery MR 750W 3T scanner using a Geometry Embracing Method coil contained within the table. Imaging consisted of 3D multi‐echo UTE Cones^17^ and 3D T1ρ mapping sequences.^18^ The multi‐echo UTE cones sequence^17^ had 21 423 conical trajectories per echo, the repetition time was 6.3 ms per trajectory, and fat suppression was applied every five trajectories. The T1rho sequence is a 3D magnetization‐prepared angle‐modulated partitioned k‐space spoiled gradient recalled acquisition that has been described previously.^18^ Parameters for the mapping sequences are summarized in Table 1. To assess in vivo repeatability of T2* mapping, subjects were scanned twice on the same day after exiting and re‐entering the scanner between scans. Subjects were scanned between 8 and 11 am, with the duration between repeat scans ranging from 15 to 30 minutes. Clinical fast spin‐echo images with T_2_ weighting (echo‐time 61.6 ms, repetition time 2500 ms, echo train length 8, acquisition matrix 256 × 256, slice thickness 3 mm) were also acquired in the sagittal orientation and used for Pfirrmann grading.

**Table 1:** Imaging parameters for human subjects and cadavers

Sequence	Parameter	Subjects	Cadavers
UTE cones	Repetition time (TR), ms	32	32
	Echo‐time (TE), ms	0.244, 5.2, 10.2, 15.2, 20.2, 25.2	0.308, 5.0, 9.7, 14.4, 19.1, 23.8, 2.7, 7.4, 12.1, 16.8, 21.5, 26.2
	Voxel, mm × mm × mm	0.5 × 0.5 × 3	0.5 × 0.5 × 3
	Field‐of‐view, cm × cm	28 × 28	28 × 28
	Flip angle, degrees	19	19
	Matrix size	560 × 560	560 × 560
T1ρ mapping	Repetition time (TR), ms	5.2	5.2
	Spin‐lock time (TSL), ms	0, 10, 40, 80	0, 2, 4, 8, 12, 20, 40, 80
	Field‐of‐view, cm × cm	20 × 20	20 × 20
	Matrix size	256 × 128	256 × 128
	Voxel, mm × mm × mm	0.78125 × 1.5625 × 8.0	0.78125 × 1.5625 × 8.0
	Number of slices	12	12

*Note*: Typical scan time: UTE approximately 13 minutes (subjects) and approximately 26 minutes (cadavers); T1ρ approximately 10 minutes.

Abbreviation: UTE, ultrashort echo‐time.

### 2.3 CEP T2* mapping

A total of 23 structurally intact CEPs (n = 16 ex vivo, n = 7 in vivo) caudal to the L4‐L5 (n = 5) or L5‐S1 (n = 18) discs were analyzed. The L4‐L5 and L5‐S1 levels were selected based on orientation criteria for imaging the CEP and measuring T2* values with UTE MRI. Specifically, the orientation of the selected CEPs with respect to the magnetic field (range: 48°‐60°) was close to 54.7°, the magic angle, thereby ensuring the resultant T2* values mainly reflect unbound water.^11^ UTE images were interpolated to a voxel size of 0.5 × 0.5 × 0.5 mm^3^. Custom software was written in IDL (Harris Geospatial Solutions) to generate CEP T2* maps. The algorithm included the following steps:

(a) CEP segmentation: we analyzed all sagittal slices that contained CEP voxels, which ranged from 51 to 88 slices depending on the CEP. On each sagittal MRI slice, the anterior and posterior ends of the CEP were located, and a contour line (thickness = 1 voxel) was created by tracing the voxels with high signal intensity (SI) between the two identified points. The resultant contour was masked over the original image, and the masked area was subsequently dilated using a voxel‐based region‐dependent inclusion criteria. For example, for a voxel C on the contour line, suppose voxel N is one of its cranially or caudally adjacent neighbors. N was included in the masked area if the following condition was met:

$$|SI_{N}-SI_{C}|\leq T_{1}\;if\;N\;is\;adjacent\;to\;the\;vertebra,\;and$$

$$|SI_{N}-SI_{C}|\leq T_{2}\;if\;N\;is\;adjacent\;to\;the\;intervertebral\;disc$$

where ${\text{SI}}_{N}$ and ${\text{SI}}_{C}$ are the SI values for N and C. Location‐specific $T_{1}$ and $T_{2}$ refer to the SI difference between CEP and bony endplate and CEP and NP, respectively, and account for the varying image intensity and contrast across the endplate.

**Figure 1:**
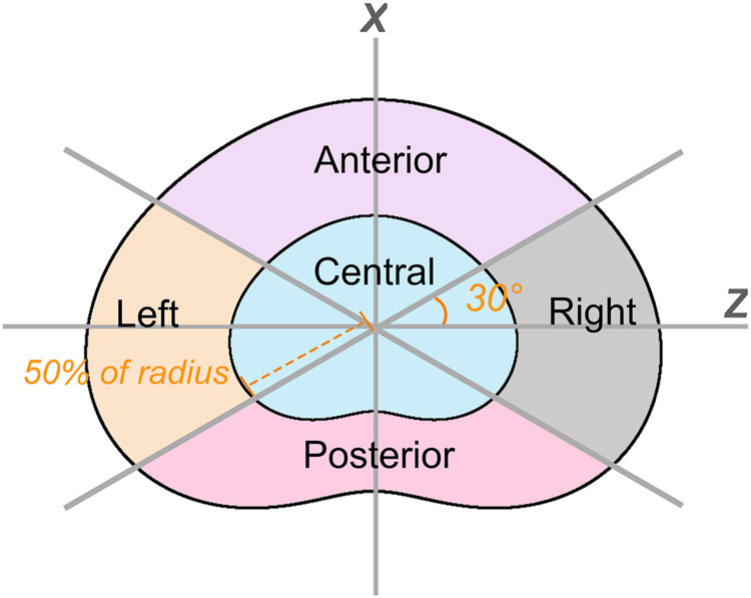
Diagram showing the standardized mapping of the CEP that was used to define subregions. The origin of the CEP was located at its area centroid. The central subregion had a radius that was 50% of the length of the outer CEP margin. The remaining area was divided into four subregions (anterior: 30°‐150°, left: 150°‐210°, posterior: 210°‐330°, right: ±30°). The number of voxels in each of the five subregions of the CEP was as follows: central approximately 580 to 880, anterior approximately 570 to 860, posterior approximately 530 to 820, left/right approximately 490 to 780. The number of slices that made up each subregion was as follows: central 25 to 44, anterior 40 to 71, posterior 46 to 80, left/right 13 to 23. CEP, cartilage endplate [Color figure can be viewed at wileyonlinelibrary.com]

(b) T2* mapping: T2* relaxation times of the segmented CEP voxels from all sagittal slices were calculated on a voxel‐by‐voxel basis by fitting the measured SI to an exponential decay function.^11^

(c) Establish coordinate system: The principal axes of the segmented CEP were identified, and the CEP was rotated so that the principal axes aligned with the x‐, y‐ and z‐axes, which coincided with the anterior‐posterior, inferior‐superior, and medial‐lateral axes, respectively.

(d) Define reference template: to map the CEP voxels into standard reference template and exclude non‐CEP voxels, Cartesian coordinates for each voxel were converted to polar coordinates. Voxels with polar coordinates outside of the area bounded by Mizrahi's equation^19^ were excluded.

(e) Define subregions: the shape of a central subregion was concentric with the outer margin of the CEP at 50% of the outer margin distance. The remaining area was divided into four subregions (anterior: 30°‐150°, left: 150°‐210°, posterior: 210°‐330°, right: ±30°; Figure 1).

To illustrate T2* maps in 2D, the CEP voxels were projected onto the z‐x plane. The mean T2* value was displayed when two or more CEP voxels projected onto one z‐x location.

### 2.4 NP T1ρ mapping

**Figure 2:**
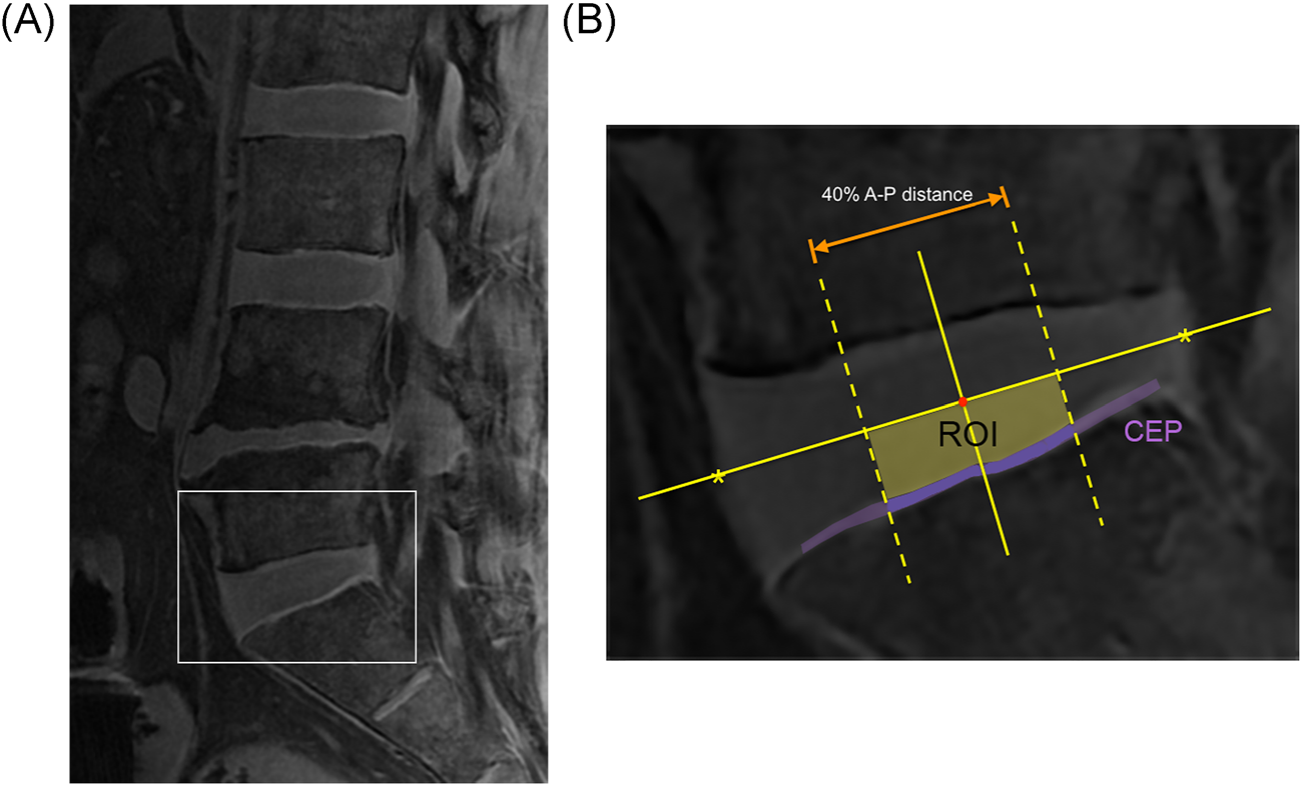
A, Midsagittal UTE image from a 53‐year‐old male showed high CEP signal intensity. B, Zoomed inset showing the location of the NP ROI for investigating the association between NP T1ρ values and CEP T2* values. ROIs for the NP had between 160 and 230 voxels. T1ρ values were averaged for NP voxels in the caudal half of the disc bordering the central CEP subregion. CEP, cartilage endplate; NP, nucleus pulposus; UTE, ultrashort echo‐time [Color figure can be viewed at wileyonlinelibrary.com]

T1ρ maps of the NP adjacent to the index CEPs were created from three midsagittal slices using voxel‐wise fitting of the signal intensities to decay curves.^18^ T1ρ values were averaged for the NP voxels in the half‐disc bordering the central CEP subregion (Figure 2).

### 2.5 Statistical analysis

Paired *t*‐tests with Holm‐Bonferroni adjustments for multiple comparisons were used to compare summary T2* parameters (mean, interquartile range) between CEP subregions. Independent associations between mean NP T1ρ, mean CEP T2* and age were quantified by the Pearson correlation coefficient. To investigate the nonmonotonic relationship between disc degeneration and CEP composition, the discs were grouped to achieve some degree of age distinction while ensuring a similar number of discs in each group. To determine “low” and “high” CEP T2* values in each age group, the CEPs within a particular age group were rank‐ordered from lowest to highest according to their mean T2* value. Then the CEPs were split into two equal‐sized groups using the median T2* value for that particular age group as the cutoff between “low” and “high” T2* values. Two‐way analysis of variance with interaction was used to test the effects of age and mean CEP T2* values on mean NP T1ρ values. Within each age group, independent two‐sample *t*tests were used to compare mean NP T1ρ values and mean CEP T2* values between discs with the “low” vs “high” mean CEP T2* values. Short‐term repeatability was estimated by the precision error, defined as the root‐mean‐square average of the precision errors in mean T2* values for the seven subjects.^20^ Reliability of T2* measurements was measured by intraclass correlation coefficient (ICC) generated from random effects models^21^ to account for repeated observations. We considered ICC values between 0.75 and 0.90 to be good reliability, and values more than 0.90 to be excellent reliability.^22^ All analyses were performed using R software (R Core Team, 2018). Data are given as mean ± standard deviation (SD).

## 3 RESULTS

### 3.1 Spatial differences in T2* values

**Figure 3:**
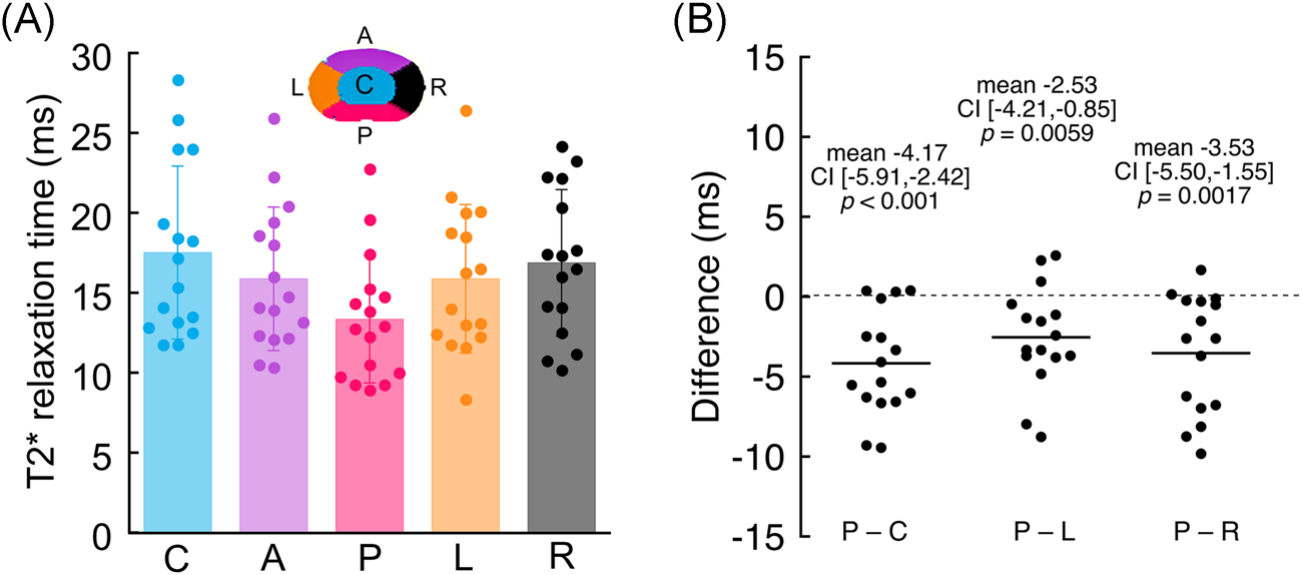
A, Mean T2* values for each subregion of *n* = 16 cadaveric CEPs imaged in situ. Mean CEP T2* values were highest centrally and lowest posteriorly. B, Pairwise differences in T2* values between CEP subregions. *P* values show the result of paired *t* tests between subregions. T2* values in the posterior subregion were significantly lower than those in the central, left, and right subregions. Pairwise differences between T2* values in the other subregions were not significant. CEP, cartilage endplate [Color figure can be viewed at wileyonlinelibrary.com]

Mean T2* values, indicative of CEP hydration and inversely related to the collagen‐to‐GAG ratio,^11^ were highest in the central CEP subregion (17.5 ± 5.4 ms) and lowest in the posterior subregion (13.4 ± 4.0 ms, Figure 3A). Mean T2* values in the posterior subregion were significantly lower than those in the central (*P* < .001), left (15.9 ± 4.7 ms, *P* = .0059) and right subregions (16.9 ± 4.6 ms, *P* = .0017, Figure 3B). The subregions had similar coefficients of variation (central: 0.31, anterior: 0.28, posterior: 0.30, left: 0.29, right: 0.27), indicating similar between‐CEP variation in mean T2* values. However, within‐CEP variation in T2* values, given by the mean interquartile range, was significantly higher in the central subregion (9.0 ± 3.4 ms) than posterior subregion (5.4 ± 1.9 ms; mean difference: 3.6; 95% confidence interval: 2.1, 5.1; *P* = .0013). The between‐CEP variability in CEP T2* values overall was 0.31 (coefficient of variation) for cadavers and 0.16 for human subjects.

### 3.2 Associations between T2*, age, and adjacent disc degeneration

**Figure 4:**
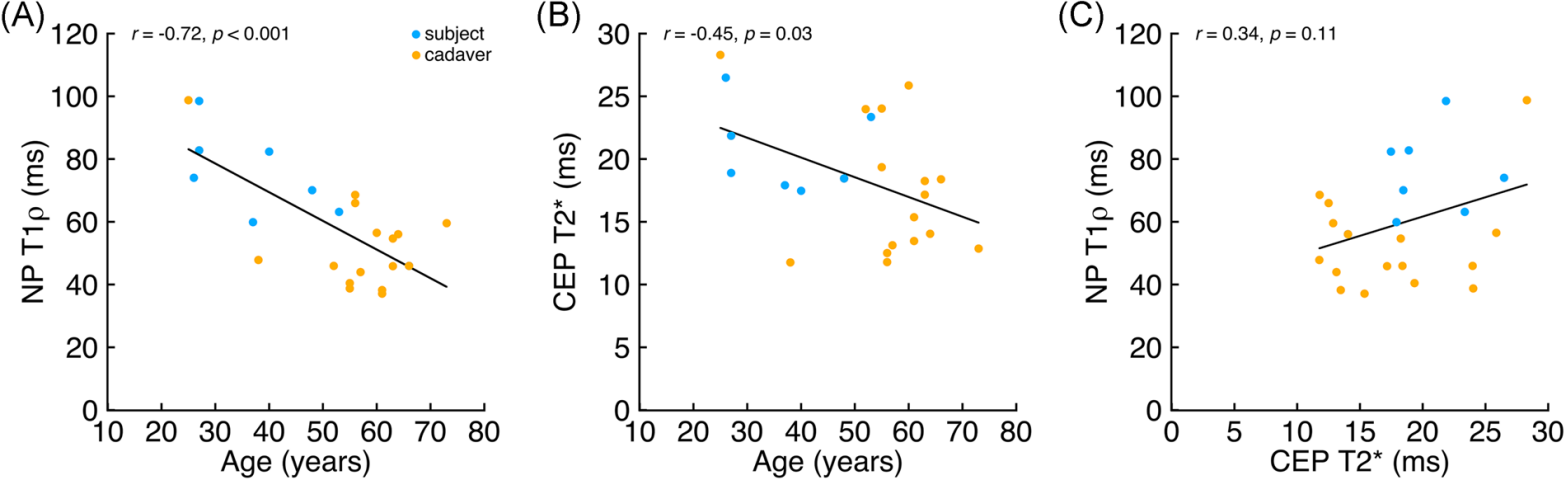
Scatterplots show relationships between NP T1ρ, CEP T2*, and age for n = 23 discs pooled. A, Age was inversely associated with mean T1ρ values in the NP. B, Age was inversely associated with mean T2* values in the central CEP. C, Mean NP T1ρ values were not significantly associated with mean T2* values in the central CEP. CEP, cartilage endplate; NP, nucleus pulposus [Color figure can be viewed at wileyonlinelibrary.com]

**Figure 5:**
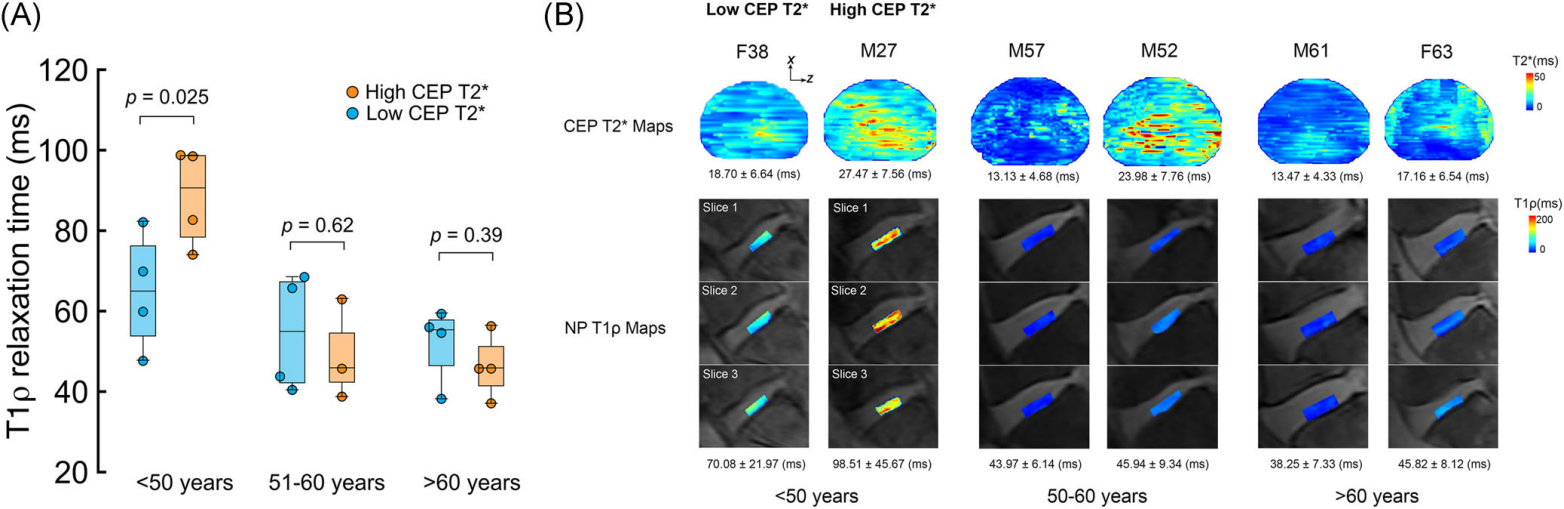
A, Discs were split into three similarly sized age groups (n = 8, 7, 8 discs). In the youngest age group (<50 years), discs that were adjacent to CEPs with the shortest T2* values had lower mean T1ρ values (*P* = .025). In the older age groups, mean NP T1ρ values were similar between discs with the highest vs lowest CEP T2* values (51‐60 years: *P* = .62; >60 years: *P* = .39). B, Representative CEP T2* maps and NP T1ρ maps (three midsagittal slices) from each age group. For ease of 2D visualization, the T2* maps were projected onto the z‐x plane. In the youngest age group (age <50 years), compared with the NP of M27, NP of F38, adjacent to CEP with shorter mean T2* relaxation time, had lower mean T1ρ value. In the older age groups, NP T1ρ values were similar for discs adjacent to CEPs with low vs high T2* values. CEP, cartilage endplate; NP, nucleus pulposus [Color figure can be viewed at wileyonlinelibrary.com]

Age was inversely associated with both mean T1ρ values in the NP (*r* = −.72, *P* < .001, Figure 4A) and mean T2* values in the central CEP (*r* = −.45, *P* = .032, Figure 4B). The relationship between NP T1ρ values and CEP T2* values for all CEPs pooled was not significant (*r* = .34, *P* = .11, Figure 4C). After splitting the discs into three similarly sized groups based on age (n = 8, 7, 8), there was a significant interaction between the effects of age and CEP T2* values on NP T1ρ values (*P* = .045), suggesting that the effect of CEP T2* values on NP T1ρ values depends on age. Specifically, in the youngest group (<50 years), the discs that were adjacent to CEPs with shorter T2* values (ie, lower CEP hydration) had 26.7% lower NP T1ρ values (*P* = .025, Figure 5 and Table 2). In the older age groups, NP T1ρ values were similar between discs with high vs low CEP T2* values (50‐60 years, *P* = .62; >60 years, *P* = .39). Also, NP T1ρ values were significantly correlated with CEP T2* values (*r* = .71, *P* = .047), but only in the youngest age group (Figure S1). The discs in the youngest age group had Pfirrmann grades II to III; in the two older age groups, grades III to IV with one grade II (Table 3).

**Table 2:** Comparison of T1ρ and T2* values between individuals with high vs low CEP T2* values

	<50 Years			51‐60 Years			>60 Years		
	Low T2* n = 4 CEP	High T2* n = 4 CEP	*P* value	Low T2* n = 4 CEP	High T2* n = 3 CEP	*P* value	Low T2* n = 4 CEP	High T2* n = 4 CEP	*P* value
NP T1ρ, ms	65.0 ± 14.7	88.5 ± 12.2	.025	54.8 ± 14.6	49.3 ± 12.6	.620	52.1 ± 9.5	46.4 ± 7.9	.390
CEP T2*, ms	16.1 ± 3.1	23.9 ± 4.3	.027	14.2 ± 3.5	23.8 ± 0.8	.010	14.7 ± 2.4	19.2 ± 4.6	.054

Abbreviations: CEP, cartilage endplate; NP, nucleus pulposus.

**Table 3:** The number of discs with each Pfirrmann grade

Pfirrmann	<50 Years		51‐60 Years		>60 Years		Total
Grade	Low T2*	High T2*	Low T2*	High T2*	Low T2*	High T2*	
Grade I	0	0	0	0	0	0	0
Grade II	1	3	1	0	0	0	5
Grade III	3	1	1	1	2	2	10
Grade IV	0	0	2	2	2	2	8
Grade V	0	0	0	0	0	0	0

### 3.3 Comparisons between cadavers and subjects

The cadavers were significantly older than the human subjects (56.4 ± 12.6 years vs 36.9 ± 56.4 years, *P* = .0019), and accordingly, the mean NP T1ρ values in the cadavers were lower (52.2 ± 16.1 vs 75.8 ± 13.3 ms, *P* = .0028). Mean T2* values were similar for the cadavers and subjects (20.6 ± 3.4 vs 17.5 ± 5.4 ms, *P* = .11).

### 3.4 Repeatability of T2* assessment in vivo

**Figure 6:**
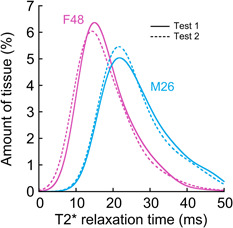
Overlapping T2* histograms show low inter‐test variability relative to inter‐subject variability. In each histogram, the amount of tissue is calculated by dividing the number of voxels with T2* values within a given range by the total number of voxels in that particular CEP. CEP, cartilage endplate [Color figure can be viewed at wileyonlinelibrary.com]

Repeat in vivo imaging and analysis indicated that inter‐test variability in the distribution of T2* values was qualitatively lower than inter‐subject variability (Figure 6). The precision error of mean T2* assessment was 1.2 ms overall (central: 2.6 ms, anterior: 1.7 ms, posterior: 1.9 ms, left: 2.5 ms, right: 2.5 ms). T2* assessment had good overall inter‐test reliability (ICC = 0.90) and good‐to‐excellent reliability in each of the five subregions (central: 0.94, anterior: 0.89, posterior: 0.91, left: 0.92, right: 0.92).

## 4 DISCUSSION

T2* relaxation times varied systematically within the CEP, suggesting that CEP composition varies regionally. Specifically, T2* values were highest centrally and lowest posteriorly. Since T2* values are positively correlated with sGAG and water contents,^11^ our new data suggest that sGAG and water contents are likewise highest centrally and lowest posteriorly. Importantly, we also discovered that inter‐subject differences in mean CEP T2* values were associated with adjacent NP T1ρ values in the youngest age group: those with lower CEP T2* values had significantly lower NP T1ρ values, indicating more severe disc degeneration.^14^, ^16^ Since the individuals in the youngest age group had mild‐to‐moderately degenerated Pfirrmann grade II to III discs, our findings suggest that deficits in CEP composition, as indicated by low T2* values, are associated with more severe disc degeneration during the mild‐to‐moderate stages. In the older groups, which mainly had severer/later Pfirrmann grade III to IV discs, NP T1ρ values were not associated with differences in adjacent CEP T2* values. Finally, to gauge feasibility of clinical T2* assessment, we evaluated in vivo repeatability. Results indicated the precision errors in mean T2* values ranged from 1.7 to 2.6 ms, and reliability was good‐to‐excellent (ICC = 0.89‐0.94). Although requiring confirmation in larger studies, our findings collectively suggest that deficits in CEP biochemical composition, as measured by UTE MRI, associate with more severe disc degeneration during the mild‐to‐moderate stages in younger individuals, and that noninvasive CEP T2* measurement could eventually allow clinicians to identify mild‐to‐moderately degenerated Pfirrmann grade II to III discs in which deficits in CEP composition may be a contributing factor.

Mean T2* values were the highest centrally and lowest posteriorly, suggesting that water and sGAG contents are likewise highest centrally and lowest posteriorly. This finding is consistent with results from previous biochemical analyses of CEP sections from three human subjects.^6^ Compared to previous studies wherein CEP tissues were sampled manually and discretely, T2* assessment provides a noninvasive, systematic, and continuous picture of the spatial variations in CEP composition that enables robust subregional comparisons. Another interesting finding is that T2* values demonstrated greater variation in the central subregion than posterior subregion. This could indicate greater compositional variation in the central subregion of the CEP. However, this could also reflect partial volume effects in the central subregion, which is known to be the thinnest region.^6^, ^9^, ^23^ Histologic measurements indicate that mean CEP thickness is 0.62 ± 0.29 mm.^6^ While the in‐plane voxel size (0.5 × 0.5 mm^2^) was smaller than the reported mean CEP thickness, and the segmented CEP masks were between 1 and 3 voxels thick (1.8 voxels on average), there may be some partial volume averaging of the boney endplate or NP that affects the interquartile range in some locations. Nevertheless, we believe any partial volume effects are likely to have a small impact on mean T2* values, since a prior study comparing histologic measures of CEP thickness to UTE measurements of CEP thickness with the same in‐plane resolution reported that the overestimation was not significantly different than zero (mean difference = 0.02 ± 0.13 mm, *P* = .32).^23^

A clinically relevant finding of this study is that low CEP T2* values coincided with greater extent of disc degeneration during the mild‐to‐moderate stages. Previous ex vivo studies found that the relationship between disc degeneration and CEP composition and permeability is nonmonotonic. Specifically, disc degeneration associated with deficits in CEP composition and permeability, but only in the earlier stages of disc degeneration,^8^, ^9^ when increased disc degeneration is thought to proceed with reduced transport. Our results here corroborate those prior findings, since low CEP T2* values correlate with lower water content and greater collagen content in the tissue,^11^ and both characteristics impact CEP transport properties. Specifically, lower water content prevents solutes from diffusing freely within the CEP,^7^, ^24^ and high collagen content hinders solute uptake and diffusion by reducing pore space.^2^, ^13^ Deficits in CEP composition and transport properties limit NP cell viability and gene expression in in vitro model systems,^2^ and our new findings provide evidence that CEP status may similarly impact disc health in younger humans in the mild‐to‐moderate stages of disc degeneration.

A second clinical implication relates to the repeatability of T2* assessment. Although deficits in CEP biochemical composition are believed to influence disc degeneration and regeneration, the composition and integrity of the CEP are not routinely evaluated in a clinical setting because CEP signal is not captured by conventional T_2_‐weighted sequences with long echo times. Our finding that mean T2* measurements from UTE MRI had good‐to‐excellent short‐term repeatability in vivo and that differences among individuals greatly exceeded differences between repeat tests indicates that T2* measurement can provide reliable, indirect assessments of CEP composition. This suggests that T2* measurement could be used to clarify the role of deficits in CEP composition in patients with Pfirrmann grade II to III discs, and by extension, T2* measurement could be used to test if CEP composition influences the response to biologic therapies meant to regenerate mild‐to‐moderately degenerated discs.

We limited our analysis to L4‐L5 and L5‐S1 CEPs because those CEPs were oriented with respect to the magnetic field at angles nearest to the magic angle. At that angle, the contributions of matrix‐bound water to T2* are minimized, and thus the measured variations in T2* values mainly reflect variations in the amount of mobile water and the collagen‐to‐GAG ratio.^11^ Although limiting T2* analysis to L4‐L5 and L5‐S1 CEPs is clearly restrictive, inferring CEP composition based on T2* values at those levels is most relevant for understanding disc degeneration and regeneration. This is because disc degeneration is most prominent at L4‐L5 and L5‐S1, and hence, those discs are frequent targets for biologic therapies. From a practical standpoint, the orientation of the L4‐L5 and L5‐S1 CEPs is near to the magic angle owing to lumbar lordosis. To help accentuate the lordotic curve and angle those CEPs more closely to the magic angle, we placed a 2‐inch‐thick foam pillow under the lumbar spine. Also, we restricted T2* analysis to CEPs that were caudal to the discs. Cranial CEPs generally had lower contrast between their adjacent tissues than caudal CEPs, thus making accurate segmentation difficult. Since caudal CEPs are less permeable than cranial CEPs,^9^ the caudal CEPs may be more likely to limit nutrient transport and could thus play a greater role in adjacent disc degeneration. That said, our findings here only apply to the caudal CEPs, and other factors, including the composition of the cranial CEP and the permeability of the bony endplate, may contribute independently.

An important limitation of this study was the relatively small number of discs, and results should be interpreted carefully and not overgeneralized. Based on the age group with the largest observed variance in mean T1ρ (SD = 13.5 ms) and a power of 0.8, the minimum detectable difference in mean T1ρ was 20.3 ms. The amount of variation in T1ρ—whether due to natural biologic heterogeneity or to noise in the MRI measurement—may necessitate larger sample sizes if more subtle differences between groups are to be detected. Related, correlation between NP T1ρ and CEP T2* values in Pfirrmann grade II to III discs suggests CEP composition as a relevant factor, and this should be validated in larger studies. A second limitation was that histologic or biochemical validation of the MRI findings was not possible. However, prior studies reported significant correlations between T1ρ values and NP GAG content^14^ and between T2* values and CEP biochemical composition.^11^ Additionally, our MRI findings are consistent with known changes in NP and CEP biochemical composition with age,^8^, ^25^ which further supports the validity of our findings. A third limitation was that the mapping sequences used for the subjects and cadavers had different echo times and spin‐lock times. However, since the echo times and spin‐lock times of the two sequences spanned similar ranges, it is unlikely that these differences affected our results. Related, we pooled in vivo and ex vivo MRI data to achieve a broad age and disc degeneration range. While subtle changes in MRI characteristics may occur postmortem, those effects appear to be small in comparison to the effects of age and disc degeneration. For example, mean NP T1ρ values for the cadavers in our study (age: 56.4 ± 12.6 years, 25‐73 years; T1ρ = 52.2 ± 16.1 ms) were similar to mean NP T1ρ values measured in vivo previously (age: 48.1 ± 14.1 years, 20‐67 years; T1ρ = 52.7 ± 15.6 ms).^16^ Also, certain exclusion criteria that could independently impact disc health, including diabetes and positive smoking status, were not applied to the cadavers and may have therefore contributed to the unexplained variability in the findings. A fourth limitation was that we tested short‐term in vivo repeatability in only seven subjects, which precludes accurate estimation of the confidence interval around the precision errors. All scans were conducted in the morning to reduce any confounding effects of diurnal variations in disc water content, and thus, repeatability over the course of daily loading remains unknown. Last, this study was cross‐sectional, and thus, we were unable to determine how changes in CEP biochemical composition relate to changes in disc health. Nevertheless, our findings here motivate T2* assessment in future longitudinal studies.

In summary, we used T2* mapping from UTE MRI to noninvasively study the biochemical composition of the CEP ex vivo and in vivo. Our findings suggest that deficits in CEP composition, as indicated by low T2* values, associate with more severe disc degeneration during the mild‐to‐moderate stages (Pfirrmann grades II‐III) in younger individuals. Future longitudinal studies will use CEP T2* assessment to clarify the role of CEP composition in disc degeneration and in patient's response to treatment.

## AUTHOR CONTRIBUTIONS

(a) Substantial contributions to research design, or the acquisition, analysis, or interpretation of data; (b) drafting of the paper or revising it critically; (c) approval of the submitted and final version.

Linshanshan Wang: a, b, c; Misung Han: a, c; Jason Wong: a, c; Patricia Zheng: a, c; Ann Lazar: a, c; Roland Krug: a, c; Aaron Fields: a, b, c.

## Supporting information

Supplementary information

Supplementary information
