# Supplementary figures and images for: Evaluation of human cartilage endplate composition using MRI: Spatial variation, association with adjacent disc degeneration, and in vivo repeatability
Source: J Orthop Res. 2020 Jul 7;39(7):1470–8. doi: 10.1002/jor.24787 (PMC7765737; doi:10.1002/jor.24787)

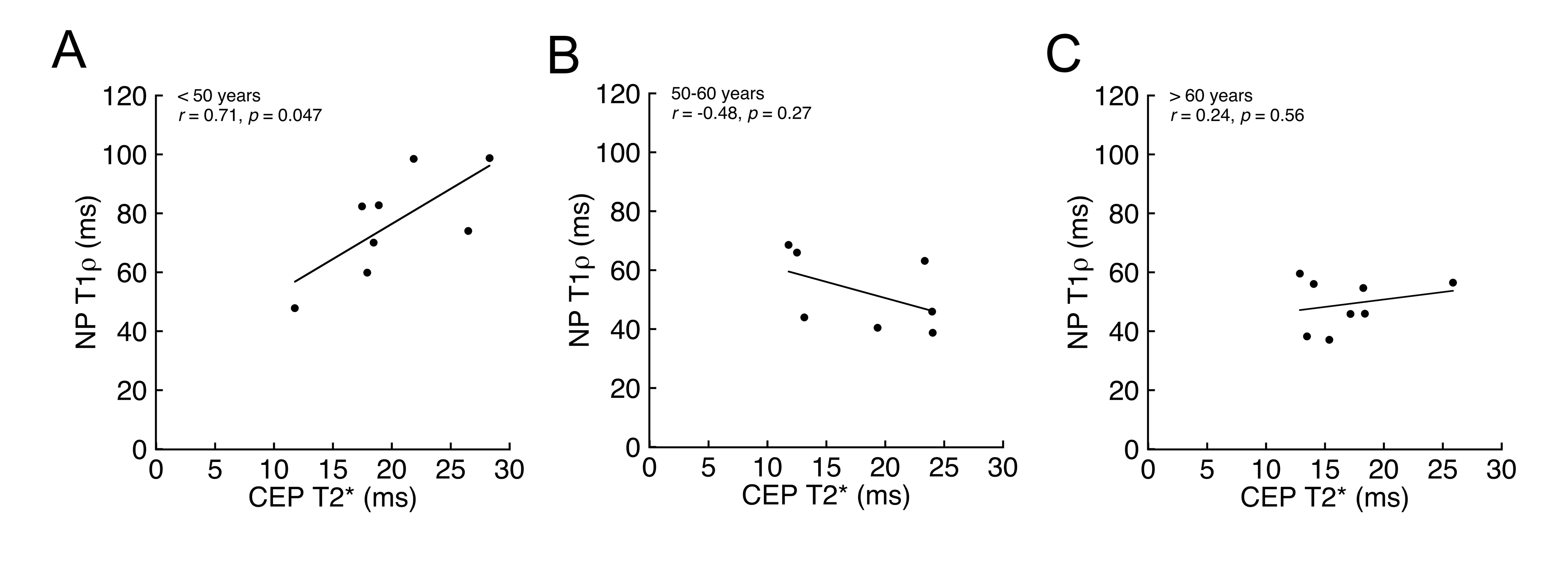

Supplement: Supplementary file 1 — Supplementary information [file JOR-39-1470-s002.tif]
