# Supplementary material for: Evaluation of human cartilage endplate composition using MRI: Spatial variation, association with adjacent disc degeneration, and in vivo repeatability
Source: J Orthop Res. 2020 Jul 7;39(7):1470–8. doi: 10.1002/jor.24787 (PMC7765737; doi:10.1002/jor.24787)
Supplement: Supplementary file 2 — Supplementary information [file JOR-39-1470-s001.docx]

**Supplementary Materials**

**Supplementary Figure S1:** Scatterplots show relationships between NP T1ρ and CEP T2* for each age group. **(A)** In the youngest age group (age < 50 years), mean T1ρ values in NP were positively correlated with mean T2* values in the central CEP. **(B, C)** In the older age groups (50-60 years; >60 years), NP T1ρ was not significantly correlated with CEP T2* values.
